# 

**Published:** 1899-07-01

**Authors:** 


					THE HOSPITAL. << The standard of highest purity."? Lancet, July 1st, 1899.
CADBURY'S rf* V to* OADBURY'S
COCOA 1L MM ..j?V COCOA
ABSOLUTELY PURE. NO DRUGS OR ALKALIES USED.
No. GGGN/VoI. XXVI. [SsTapen] Saturday>
July 1st, 1899.
[SubscripH?nTermSlJ pBiICE TWOPENCE.

				

